# Effects of broiler breeder age on the performance, nutrient metabolism, and intestinal histomorphometry of broiler chickens

**DOI:** 10.1007/s11250-026-05051-4

**Published:** 2026-05-08

**Authors:** Helder Freitas de Oliveira, Alessandra Gimenez Mascarenhas, José Henrique Stringhini, Adriana Santana do Carmo, Marcos Barcellos Cafe, Raphael Rodrigues dos Santos, Imar Crisógno Fernandes Filho, Deibity Alves Cordeiro, Jean Kaique Valentim, Heloisa Helena de Carvalho Mello

**Affiliations:** 1 Department of Animal Science, Federal University of Goiás (UFG), Goiânia, 74.690-900 Brazil; 2 Department of Animal Production, Federal Rural University of Rio de Janeiro (UFRRJ), Seropédica, 23.897-000 Brazil

**Keywords:** Broiler, Breeder age, Early growth, Gut development, Nutrient metabolism

## Abstract

An experiment was conducted to evaluate the effects of broiler breeder age on the growth performance, nutrient metabolism, and intestinal histomorphometry of the progeny during early life. A total of 180 one-day-old Cobb 500^®^ chicks, with an average initial body weight of 45.82 ± 2.21 g, were assigned to a completely randomized design with three treatments, six replicates, and 10 birds per replicate. The treatments consisted of chicks derived from broiler breeders aged 39, 51, and 69 weeks. Productive performance, gastrointestinal tract histomorphometry, and nutrient metabolism were evaluated. Chicks originating from older breeders (69 weeks) presented superior growth performance, whereas those from younger breeders (39 weeks) presented greater nutrient metabolism. With respect to intestinal morphology, progeny from older breeders presented more developed intestinal structures at 1 and 21 days of age, whereas at 7 days of age, chicks from younger breeders presented more pronounced intestinal morphometric characteristics. Broiler breeder age influences the early performance, intestinal development, and nutrient metabolism of progeny. Older breeders favor early growth and intestinal maturation, whereas younger breeders enhance protein metabolism, highlighting the relevance of breeder age in early broiler management. These findings provide integrated evidence linking breeder age to growth performance, intestinal development, and nutrient metabolism during early life.

## Introduction

The global population has grown exponentially in recent decades, resulting in increased demand for animal-derived foods. In this context, broiler chickens have become one of the main sources of high-quality protein, providing essential nutrients while being produced efficiently within a short production cycle and at a relatively low cost (Mottet and Tempio 2017). According to Borges et al. (2021), the poultry industry has experienced continuous growth, with increasing production and commercialization of meat and related products. Advances in genetic selection, animal welfare, housing and environmental management, nutrition, and the development of new technologies have been decisive factors for the consolidation of broiler production systems (Brassó et al. 2025).

To ensure the competitiveness and sustainability of this sector, proper evaluation of broiler breeder production is essential (Ramukhithi et al. 2023). The industrial production of day-old chicks represents a strategic segment within the poultry industry and plays a critical role in the success of the production chain. Egg incubation, which transforms fertile eggs into day-old chicks, marks the beginning of the broiler production cycle, and breeder age is one of the main factors affecting hatchery productivity (Tona et al. 2022). Currently, the increasing demand for day-old chicks has led to extended breeder flock cycles, resulting in a greater proportion of eggs produced by older breeders (Mesele 2023).

Genetic improvement programs have therefore focused on increasing laying persistency, which may directly affect the quality of hatching eggs and day-old chicks, ultimately influencing broiler performance throughout the production cycle (Molenaar et al. 2023). Broiler production at all operational levels depends fundamentally on a consistent supply of high-quality day-old chicks (Köseman et al. 2020).

Artificial incubation enables the efficient production of large numbers of chicks, and the incubation period accounts for approximately 30% of the total broiler production cycle (Gonçalves et al. 2013). The quality of chicks delivered to farms is a key determinant of productive success, as performance parameters and intestinal histomorphometric development are closely associated with feed efficiency (Rysman et al. 2023). Therefore, factors that influence chick quality during embryonic development, such as breeder age, may have important consequences for early growth and physiological development.

Intestinal development during the early life of broiler chickens plays a crucial role in determining nutrient digestion and absorption capacity. According to Uni et al. (1996), the intestinal morphology of chicks markedly changes between 4 and 21 days of age, characterized by increases in villus volume and crypt depth, which reflect increased absorptive capacity. Subsequent studies confirmed that posthatch epithelial development varies among intestinal segments, with the duodenum showing rapid villus development by 7 days of age, whereas the number of villi in the jejunum and ileum continues to increase until approximately 14 days post-hatch (Uni et al. 1998).

Birds with greater absorptive capacity, associated with increased villus height and density, generally show improved feed efficiency. Maiorka et al. (2016) reported that embryos derived from older breeders presented significantly taller villi in the duodenum, jejunum, and ileum; however, the villus density was greater in embryos from younger breeders. Similarly, Fernandes et al. (2014) reported that chicks from younger breeders exhibit deeper intestinal crypts than those from older breeders, which may directly affect nutrient utilization. These findings indicate that breeder age may influence intestinal morphology and nutrient utilization during early life.

Although previous studies have demonstrated that breeder age influences chick weight and early performance, limited information is available regarding the combined effects of breeder age on intestinal histomorphometry and nutrient metabolism during early broiler development. Understanding these physiological responses is essential for optimizing early nutrition and management strategies in commercial broiler production systems. However, studies that integrate growth performance, intestinal histomorphometry, and nutrient metabolism within a single experimental framework, especially under real-world production conditions, remain scarce.

We hypothesize that breeder age differentially affects intestinal morphology, nutrient metabolism, and growth performance of broiler chickens during the early growth phase. Therefore, the objective of this study was to evaluate the effects of broiler breeder age on nutrient metabolism, growth performance, and intestinal histomorphometry in broiler chickens.

## Materials and methods

The experiment was conducted at the Experimental Poultry Facility of the Department of Animal Science, School of Veterinary and Animal Science, Federal University of Goiás (UFG), Goiânia, Goiás, Brazil. All experimental procedures were approved by the Ethics Committee on the Use of Animals (CEUA/UFG) under protocol no. 076/18.

A total of 180 one-day-old Cobb 500^®^ broiler chicks, with an average initial body weight of 45.82 ± 2.21 g, were used in the performance trial. In addition, two extra chicks from the same breeder flocks were used exclusively for intestinal histomorphometric analysis at day 1 and were not included in the experimental units.

After the initial sampling at day 1, birds were allocated to a completely randomized design with three treatments, six replicates, and 10 birds per replicate. Birds were sexed at placement using the feathering method, and each replicate was initially composed of five males and five females.

The treatments were defined according to the age of the broiler breeders from which the chicks originated: 39, 51, and 69 weeks. The experimental period lasted 25 days and was divided into two performance phases (1–7 and 8–21 days), followed by a metabolizability assay (21–25 days).

The birds were reared on floor pens (2.10 × 2.50 m), totaling 18 experimental units, equipped with pendulum drinkers and feeders, and littered with rice husks. Pens were located inside a masonry poultry house with clay tile roofing, concrete floors, wire-mesh sidewalls, a negative ventilation system, infrared heating lamps, and misting devices. Feed and water were provided *ad libitum* throughout the experimental period. The environmental temperature and relative humidity were monitored daily, and continuous lighting was provided via infrared lamps.

All birds received the same basal diet based on corn and soybean meal, supplemented with crystalline amino acids and vitamin–mineral premixes, formulated to meet the nutritional requirements of Cobb 500^®^ broilers according to Rostagno et al. (2017). The diet compositions and calculated nutrient levels for the prestarter and starter phases are presented in Table 1.

**Table 1:** Ingredient composition and calculated nutrient levels of the experimental diets

Ingredient composition (%)	Phases	
	1–7 days	8–21 days
Corn	54.491	56.381
Soybean meal	37.018	34.854
Dicalcium phosphate	1.907	1.682
Soybean oil	1.473	2.234
Limestone	0.907	0.829
Salt	0.442	0.428
L-Lysine	1.545	1.475
DL-Methionine	0.806	0.761
Premix^1^	0.400	0.400
**TOTAL**	**100.000**	**100.000**
**Nutrients composition**		
Metabolizable energy (kcal/kg)	2.975	3.050
Crude protein (%)	24.27	23.31
Calcium (%)	0.971	0.878
Available phosphorus (%)	0.463	0.419
Sodium (%)	0.225	0.218
Digestible lysine (%)	1.307	1.256
Digestible Met + Cys (%)	0.967	0.929
Digestible threonine (%)	0.863	0.829
Digestible tryptophan (%)	0.235	0.226

¹Vitamin–mineral premix: guaranteed levels per kilogram of product: vitamin A, 1,750,000 IU; vitamin D₃, 500,000 IU; vitamin E, 3,000 IU; vitamin K₃, 375 mg; vitamin B₁, 400 mg; vitamin B₂, 1,250 mg; vitamin B₆, 750 mg; vitamin B₁₂, 2,500 µg; folic acid, 250 mg; pantothenic acid, 3,250 mg; niacin, 8,000 mg; biotin, 2.5 mg; choline, 80 g; iron, 10 g; copper, 2,500 mg; manganese, 20 g; zinc, 18 g; iodine, 250 mg; selenium, 75 mg; bacitracin, 13.75 g; sodium salinomycin, 16.5 g

At 7 and 21 days of age, growth performance parameters, including feed intake (g), body weight (g), weight gain (g), and feed conversion ratio (kg/kg), were evaluated. Feed intake was calculated as the difference between the feed offered and the feed refused for each experimental unit. Body weight was determined by dividing the total pen weight by the number of birds in each unit. Weight gain was calculated as the difference between the final and initial body weights. Feed conversion ratio (FCR) was calculated as the ratio of feed intake to body weight gain and adjusted according to the number of birds present in each experimental unit over time, considering the planned removal of birds at 7 and 21 days of age. Performance from 1 to 7 days was calculated based on the initial number of birds, whereas performance from 8 to 21 days considered the reduced number of birds after sampling.

For intestinal histomorphometric analysis, sampling at day 1 was performed using additional birds that were not part of the experimental units. At 7 and 21 days of age, two birds per replicate were randomly selected from each experimental unit, ensuring that selected birds had body weights within ± 10% of the average weight of the respective experimental unit. Thus, the number of birds per replicate was reduced from 10 to 8 (at day 7) and from 8 to 6 birds (at day 21). This reduction in bird number per replicate was inherent to the sampling procedure and occurred uniformly across all treatments. Performance variables were calculated considering the actual number of birds present in each experimental unit at each evaluation period.

The birds were fasted for six hours, weighed, and euthanized by cervical dislocation. Samples of the duodenum, jejunum, and ileum were collected, and 5-cm segments from each portion were excised for analysis of villus height, crypt depth, and the villus-to-crypt ratio.

The samples were longitudinally opened, fixed on polystyrene boards, and immersed in 10% buffered formalin for 24 h. Subsequently, the samples were stored in 70% ethanol and sent to the Histopathology Laboratory of the Department of Veterinary Medicine for slide preparation. Histological processing followed the methodology described by Luna (1968), and the sections were stained with hematoxylin and eosin (H&E). Images were obtained at 5× magnification using a Leica DM 4000B optical microscope coupled to a computer. Morphometric measurements were performed via ImageJ software. Villus height was measured from the upper base of the crypt to the villus tip, the crypt depth from the lower base to the upper base of the crypt, and the villus-to-crypt ratio was calculated accordingly.

After the performance phase (1 to 21 days), the remaining six birds per replicate were used in the metabolizability trial, totaling 108 Cobb 500^®^ broilers (54 males and 54 females) at 21 days of age, with an average initial body weight of 645.58 ± 42.23 g. Birds were distributed in a completely randomized design with three treatments, six replicates, and six birds per replicate, according to broiler breeder age (39, 51, and 69 weeks).

The birds were housed in 18 galvanized steel metabolic cages (0.40 × 0.50 m) equipped with trough feeders, drinkers, and excreta collection trays lined with plastic sheets. The metabolizability assay was conducted from 21 to 25 days of age using the total excreta collection method, as described by Sakomura and Rostagno (2016). Excreta were collected twice daily, placed in identified plastic bags, and stored at − 20 °C.

The samples were pre-dried in a forced-air oven at 55 ± 5 °C, ground in a Wiley mill, and analyzed according to Silva and Queiroz (2002). Nutrient metabolizability coefficients were calculated following classical procedures (Matterson et al. 1965; Batal and Parsons 2002; Noy and Sklan 2002). Nitrogen balance was calculated as the difference between nitrogen intake and nitrogen excretion, based on feed intake and total excreta collection, as described by Sakomura and Rostagno (2016). Metabolizability coefficients were expressed as the percentage of retained nutrients and energy relative to intake.

### Statistical analysis

Data were initially evaluated for normality of residuals via the Shapiro–Wilk test and homogeneity of variances via Levene’s test. After the assumptions were met, the data were subjected to analysis of variance (ANOVA). When significant effects were detected, the means were compared via Tukey’s test at a significance level of α = 0.05. Statistical analyses were performed via R software (R Core Team 2021). The statistical model adopted was as follows:

$$\:{Y}_{ij}=\mu+{A}_{i}+{\varepsilon}_{ij}$$

where Y_ij_ is the observed value of the dependent variable, µ is the overall mean, A_i_ is the fixed effect of breeder age (i = 39, 51, and 69 weeks), and Ɛ_ij_ is the random error associated with the observation.

## Results

Broiler breeder age significantly affected broiler performance from 1 to 7 days of age (*P* < 0.05). Chicks derived from younger breeders (39 weeks) presented lower final body weights and lower feed intakes, whereas chicks from older breeders (69 weeks) presented higher final body weights and higher feed intakes. The variables of the birds from breeders aged 51 weeks did not differ from those of the other treatments. No significant effects of breeder age were detected for the remaining performance variables or for the cumulative period from 1 to 21 days of age (*P* > 0.05) (Table 2). No mortality was observed during the experimental period.

**Table 2:** Growth performance (final body weight, weight gain, feed intake, and feed conversion ratio) of broilers from breeders of different ages from 1 to 7 and 1 to 21 days of age

Breeder age (weeks)	FBW (g)	WG (g)	FI (g)	FCR (kg/kg)
1–7 days of age				
39	133.1 b	92.2	116.8 b	1.301
51	146.7 ab	100.5	128.0 ab	1.276
69	149.5 a	101.6	133.2 a	1.313
P value	0.019	0.096	0.022	0.647
SEM	3.786	3.838	3.521	0.028
CV (%)	6.12	9.23	6.81	5.43
1–21 days of age				
39	631.4	495.7	588.0	1.689
51	643.0	496.3	596.9	1.631
69	662.4	512.9	614.5	1.658
P-value	0.465	0.805	0.557	0.764
SEM	17.443	19.378	17.283	0.056
CV (%)	6.62	12.14	7.06	8.27

Means followed by different letters within a column differ by Tukey’s test (*P* < 0.05). SEM = standard error of the mean; CV = coefficient of variation. *n* = 6 replicates per treatment (10 birds per replicate)

Breeder age significantly influenced the intestinal histomorphometry of one-day-old chicks (*P* < 0.05). Progeny from older breeders (69 weeks) presented greater villus height in all segments of the gastrointestinal tract. However, in the duodenum, these values were similar to those observed in chicks from 51-week-old breeders, whereas in the ileum, they were similar to those from 39-week-old breeders. Crypt depth in the duodenum was greater in chicks from 39-week-old breeders than in those from 69-week-old breeders. In the jejunum, chicks from older breeders presented greater crypt depth, whereas no effect of breeder age was observed in the ileum. The villus height‒crypt depth ratio was greater in chicks from older breeders across all intestinal segments, although similar values were observed between chicks from 39- and 69-week-old breeders in the ileum (Table 3).

**Table 3:** Villus height, crypt depth, and villus-to-crypt ratio in the duodenum, jejunum, and ileum of broiler chickens at one, 7 and 21 days of age from breeders of different ages

Variables		Breeder age (weeks)			*P*-value	SEM	CV
		39	51	69			
one-day-old							
Duodenum	Villus height (µm)	517.5 b	572.8 a	573.0 a	0.012	14.812	13.36
	Crypt depth (µm)	609.2 a	570.0 ab	505.8 b	0.002	1.993	17.74
	Villus-to-crypt ratio	8.7 b	10.3 ab	12.0 a	< 0.001	0.547	26.50
Jejunum	Villus height (µm)	361.5 b	350.7 b	609.5 a	< 0.001	21.039	23.88
	Crypt depth (µm)	502.3 b	483.4 b	583.3 a	0.001	1.976	18.89
	Villus-to-crypt ratio	7.4 b	7.6 b	10.6 a	< 0.001	0.495	28.93
Ileum	Villus height (µm)	340.7 a	249.6 b	325.5 a	< 0.001	10.187	16.69
	Crypt depth (µm)	609.1	607.2	558.7	0.351	2.770	23.41
	Villus-to-crypt ratio	6.1 a	4.2 b	6.0 a	< 0.001	0.331	30.63
7-day-old							
Duodenum	Villus height (µm)	823.1 ab	895.5 a	753.8 b	< 0.001	24.118	14.63
	Crypt depth (µm)	159.9	171.2	167.6	0.286	5.117	15.39
	Villus-to-crypt ratio	5.3	5.3	4.7	0.089	0.240	23.57
Jejunum	Villus height (µm)	785.0	825.8	769.8	0.148	20.710	13.05
	Crypt depth (µm)	190.1 a	177.2 a	146.4 b	< 0.001	5.935	17.33
	Villus-to-crypt ratio	4.2 b	4.8 ab	5.4 a	< 0.001	0.198	20.68
Ileum	Villus height (µm)	501.8 a	297.1 b	463.0 a	< 0.001	18.508	22.00
	Crypt depth (µm)	138.0 a	127.5 a	111.2 b	< 0.001	3.246	12.93
	Villus-to-crypt ratio	3.7 b	2.4 c	4.2 a	< 0.001	0.163	23.72
21-day-old							
Duodenum	Villus height (µm)	1202.5 b	1068.3 c	1459.7 a	< 0.001	35.270	14.18
	Crypt depth (µm)	247.1	232.7	233.9	0.168	5.905	12.41
	Villus-to-crypt ratio	4.9 b	4.6 b	6.4 a	< 0.001	0.216	20.35
Jejunum	Villus height (µm)	727.2 b	1203.3 a	1274.1 a	< 0.001	44.550	20.85
	Crypt depth (µm)	239.2 a	206.1 b	229.1 ab	0.016	8.115	18.05
	Villus-to-crypt ratio	3.1 b	5.9 a	5.7 a	< 0.001	0.205	20.95
Ileum	Villus height (µm)	936.4 b	931.9 b	1351.2 a	< 0.001	31.138	14.51
	Crypt depth (µm)	225.3	211.4	203.5	0.238	9.160	21.46
	Villus-to-crypt ratio	4.4 b	4.5 b	6.8 a	< 0.001	0.222	21.32

Means followed by different letters within a row differ by Tukey’s test (*P* < 0.05). SEM – Standard error of the mean; CV – Coefficient of variation

*n* = 6 replicates per treatment; two birds per replicate were sampled for histomorphometric analysis

At seven days of age, breeder age continued to affect intestinal histomorphometry (*P* < 0.05). Chicks from 51-week-old breeders presented greater villus height in the duodenum, whereas those from 69-week-old breeders presented the lowest values. No differences in jejunal villus height were detected among the treatments. In the ileum, chicks from 39- and 69-week-old breeders presented greater villus height. Crypt depth in the duodenum was not affected by breeder age; however, deeper crypts were observed in the jejunum and ileum of chicks from 39- and 51-week-old breeders. The VH-to-crypt depth ratio did not differ in the duodenum but was greater in the jejunum and ileum of chicks from older breeders, with intermediate values observed in chicks from 51-week-old breeders (Table 3).

At 21 days of age, breeder age significantly affected intestinal morphology (*P* < 0.05). Chicks from 69-week-old breeders presented greater villus height in all intestinal segments. In the duodenum, chicks from 51-week-old breeders presented the lowest villus height, followed by those from 39-week-old breeders. In the jejunum, chicks from 51- and 69-week-old breeders presented similar villus heights, both of which were greater than those of younger breeders. In the ileum, the villus height was lower in chicks from 39- and 51-week-old breeders. Crypt depth was not affected in the duodenum or ileum; however, in the jejunum, chicks from 39-week-old breeders presented deeper crypts. The VH-to-crypt depth ratio was greater in the duodenum and ileum of chicks from older breeders than in the jejunum of chicks from 51- and 69-week-old breeders (Table 3).

Breeder age also influenced nutrient metabolism (*P* < 0.05). Chicks from younger breeders (39 weeks) presented a greater coefficient of crude protein metabolism. For mineral matter metabolism, chicks from 39- and 69-week-old breeders presented higher coefficients than did those from 51-week-old breeders (Table 4).

**Table 4:** Metabolizability coefficients and nitrogen balance of broilers from breeders of different ages determined by total excreta collection

Breeder age (weeks)	Dry matter metabolizability (%)	Crude protein metabolizability (%)	Ether extract metabolizability (%)	Ash metabolizability (%)	Nitrogen balance (g)
39	76.42	59.45 a	80.87	38.95 a	38.55
51	76.35	53.25 b	83.80	25.85 b	31.87
69	74.17	53.37 b	82.52	36.25 a	35.05
P-value	0.061	0.019	0.073	< 0.001	0.251
SEM	0.648	1.407	0.778	1.663	2.626
CV(%)	1.71	5.08	1.89	9.88	14.94

Means followed by different letters within a column differ by Tukey’s test (*P* < 0.05). SEM = standard error of the mean; CV = coefficient of variation. *n* = 6 replicates per treatment (6 birds per replicate)

## Discussion

Broiler breeder age markedly influenced the early growth performance and intestinal development of the progeny, particularly during the first week of life. The lower body weight and feed intake observed in chicks from younger breeders during this period are attributed mainly to reduced hatch weight and less advanced gastrointestinal tract development, which limit physical feed intake capacity and delay early growth (Maiorka 2002). In contrast, chicks from older breeders, which originate from heavier eggs, generally present greater nutrient demands and feed intake to support accelerated growth, as energy and protein requirements increase with body weight (Meurer et al. 2008).

These results are consistent with previous studies reporting inferior early performance in chicks derived from younger breeders and superior initial growth in progeny from older breeders (El-Sabry et al. 2015; Castro et al. 2020). However, the absence of performance differences up to 21 days of age suggests the occurrence of compensatory growth, whereby chicks from younger breeders overcome initial disadvantages owing to the high growth potential characteristic of the starter phase (Tona et al. 2004; Meurer et al. 2008).

The superior intestinal development observed in progeny from older breeders across the evaluated ages indicates enhanced absorptive capacity, as evidenced by greater villus height and a greater VH-to-crypt depth ratio. Intestinal maturation is strongly influenced by nutrient availability during embryogenesis, particularly lipids from the yolk and proteins from the albumen, which are present in greater amounts in eggs laid by older breeders (Uni et al. 1996; Gonçalves et al. 2013). Studies have shown that embryos and chicks from older breeders exhibit more advanced intestinal mucosal development, including longer villi and deeper crypts, from late incubation through early posthatching, favoring nutrient digestion and absorption (Maiorka et al. 2016; Cardeal et al. 2022).

From a physiological perspective, increased villus height combined with moderate crypt depth reflects a more mature intestinal epithelium with improved absorptive efficiency and controlled epithelial turnover (Uni et al. 1998; Kuzmuk et al. 2005). This morphological pattern represents metabolic adaptation to increased nutrient uptake and is directly associated with improved early growth performance (Gomes et al. 2007). The present findings corroborate previous reports demonstrating enhanced intestinal development in chicks derived from older breeders (Maiorka et al. 2000; Agostinho et al. 2012; Leandro et al. 2017; Machado et al. 2020).

Despite the superior intestinal morphology observed in chicks from older breeders, higher crude protein metabolism was detected in progeny from younger breeders. This response may be associated with deeper intestinal crypts, indicative of increased epithelial renewal, which can enhance the digestion and absorption of specific nutrients during early life (Fernandes et al. 2014). Nevertheless, the metabolizability coefficients observed in this study were lower than those typically reported for commercial broiler systems, possibly reflecting differences in management conditions and physiological maturity (Luegas et al. 2015; Fernandes et al. 2015; Borsatti et al. 2016).

Additionally, the absence of vaccination in the present study may have influenced nutrient utilization. Immune system development begins during embryogenesis and plays a crucial role in posthatch performance. Insufficient maternal immune protection may increase competition between the intestinal microbiota and the host for nutrients, thereby negatively affecting performance and metabolism (Baurhoo et al. 2009).

Overall, the results indicate that older breeders have advantages in terms of early intestinal development and initial performance, likely due to improved egg nutrient composition and more advanced embryonic gastrointestinal maturation. Conversely, chicks from younger breeders appear to rely on compensatory mechanisms, particularly protein metabolism, to support growth during early life.

## Conclusions

Broiler breeder age significantly influences early intestinal development and nutrient utilization in broiler chickens. Progeny from older breeders showed more advanced intestinal morphology and greater villus development, which may contribute to improved absorptive capacity during early life. Conversely, chicks from younger breeders presented greater crude protein metabolizability, suggesting differences in digestive efficiency associated with intestinal epithelial turnover. These findings highlight the importance of considering breeder age when establishing early nutritional and management strategies in broiler production systems.

## Data Availability

The datasets generated and/or analyzed during the current study are not publicly available because only the processed data (means and results of the statistical analyses) are presented in the manuscript. However, the raw data that support these findings are available from the corresponding author upon reasonable request.

## References

[CR1] Agostinho TSP, Calixto LFL, Gomes AVCG, Togashi CK, Curvello FA, Lima MF (2012) Development of gastrointestinal tract organs and performance of broiler chickens fed during the preplacement phase. Rev Bras Saúde Prod Anim 13(4):1143–1155. 10.1590/S1519-99402012000400015

[CR2] Batal AB, Parsons CM (2002) Effects of age on nutrient digestibility in chicks fed different diets. Poult Sci 81(3):400–407. 10.1093/ps/81.3.400 11902418 10.1093/ps/81.3.400

[CR3] Baurhoo B, Ferket PR, Zhao X (2009) Effects of diets containing different concentrations of mannanoligosaccharide or antibiotics on growth performance, intestinal development, cecal and litter microbial populations, and carcass parameters of broilers. Poult Sci 88(11):2262–2272. 10.3382/ps.2008-00562 19834074 10.3382/ps.2008-00562

[CR4] Borges KM, Mello HHC, Café MB, Arnhold E, Xavier HPF, Oliveira HF, Mascarenhas AG (2021) Effect of dietary inclusion of guanidinoacetic acid on broiler performance. Rev Colomb Cienc Pecu 34(2):95–104. 10.17533/udea.rccp.v34n2a02

[CR5] Borsatti L, Nunes RV, Schone RA, Frank R, Schneiders JL, Savoldi TL (2016) Nutrient digestibility in broiler diets supplemented with growth promoters. Arq Bras Med Vet Zootec 68(1):201–207. 10.1590/1678-4162-8033

[CR6] Brassó LD, Komlósi I, Várszegi Z (2025) Modern technologies for improving broiler production and welfare: A review. Animals 15(4):493. 10.3390/ani15040493 40002975 10.3390/ani15040493 PMC11851384

[CR8] Cardeal PC, Araújo ICS, Vaz DP, Abreu ARC, Melo EF, Saldanha MM, Lara LJC (2022) Effects of breeder age and preplacement feed on IgY concentration in egg yolk and chick serum. J Anim Physiol Anim Nutr 106(3):561–565. 10.1111/jpn.13604 10.1111/jpn.13604 34231928

[CR7] Castro RMA, Carvalho FB, Stringhini JH, Café MB, Oliveira EM, Jardim Filho RM, Jardim MM (2020) Breeder age and fertile egg weight on early development of chicks fed micropelleted and ground prestarter diets. Braz J Anim Environ Res 3(3):1600–1615. 10.34188/bjaerv3n3-077

[CR9] El-Sabry MI, Yalçin S, Turgay-İzzetoğlu G (2015) Effect of breeder age and lighting regimen on growth performance, organ weights, villus development, and bursa of Fabricius histological structure in broiler chickens. Czech J Anim Sci 60(3):116–122. 10.17221/8076-CJAS

[CR10] Fernandes JIM, Contini JP, Scapini LB, Gurski TJ, Esser AFG, Santos AL (2014) Influence of breeder age on organ biometry and small intestine mucosa morphometry of chicks at hatch. Semina Cienc Agrar 35(2):1083–1090. 10.5433/1679-0359.2014v35n2p1083

[CR11] Fernandes RTV, Arruda AMV, Araújo MS, Melo AS, Marinho JBM, Vasconcelos NVB, Lopes FF, Holanda JS (2015) Energy values and digestibility coefficients of a traditional diet for Label Rouge chickens at different ages. Acta Vet Bras 9(2):108–113. 10.21708/avb.2015.9.2.4255

[CR37] Gomes JDF, Putrino SM, Martinelli MR, Ishi MP, Sobral PJA, Fukushima RS (2007) Morfologia de órgãos digestivos e não digestivos de suínos de linhagens modernas durante as fases de crescimento terminação e pós-terminação. Acta Scientiarum. Anim Sci 29(3):261–266. 10.4025/actascianimsci.v29i3.553

[CR12] Gonçalves FM, Santos VL, Contreira CL, Farina G, Kreuz BS, Gentilini FP, Anciuti MA, Rutz F (2013) In ovo nutrition: A strategy for precision nutrition in poultry production systems. Arch Zootec 62(237):45–55. 10.21071/az.v62i237.1956

[CR13] Köseman A, Akdemir F, Şeker I (2020) Effects of chitosan coating and different storage periods of broiler breeder eggs on growth performance and carcass characteristics. Rev Bras Zootec 49:e20190282. 10.37496/rbz4920190282

[CR14] Kuzmuk KN, Swanson KS, Tappenden KA, Schook LB, Fahey GC Jr (2005) Diet and age affect intestinal morphology and large bowel fermentative end-product concentrations in senior and young adult dogs. J Nutr 135(8):1940–1945. 10.1093/jn/135.8.1940 16046720 10.1093/jn/135.8.1940

[CR15] Leandro NSM, Gomes NA, Café MB, Carvalho FB, Stringhini JH, Laboissière M (2017) Lymphoid organ histomorphometry and intestinal development of broiler chicks from breeders of different ages subjected to heat stress during incubation. Cienc Anim Bras 18:e34828. 10.1590/1089-6891v18e-34828

[CR16] Luegas JAP, Albino LFT, Tavernari FC, Barros VRSM, Pessoa GBS, Rostagno HS (2015) Effect of probiotic supplementation on ileal digestibility of dry matter and protein in broilers. Arch Zootec 64(247):1–7. 10.21071/az.v64i247.410

[CR17] Luna LG (1968) Manual of histologic staining methods of the Armed Forces Institute of Pathology, 3rd edn. McGraw-Hill, New York, NY

[CR18] Machado JP, Mesquita MA, Café MB, Assis SD, Veríssimo S, Santos RR, Leandro NSM, Araújo ICS (2020) Effects of breeder age on embryonic development, hatching results, chick quality, and growing performance of the slow-growing genotype. Poult Sci 99:6697–6704. 10.1016/j.psj.2020.09.008 33248585 10.1016/j.psj.2020.09.008 PMC7704964

[CR21] Maiorka A (2002) Effect of breeder age and trophic agent (glutamine) on intestinal mucosa development and pancreatic enzymatic activity of broiler chicks during the first week of life. PhD Dissertation, Universidade Estadual Paulista, Jaboticabal, Brazil

[CR19] Maiorka A, Santin E, Silva AVF, Bruno LDG, Boleli IC, Macari M (2000) Gastrointestinal tract development of embryos from broiler breeders aged 30 and 60 weeks. Braz J Poult Sci 2(2):141–148. 10.1590/S1516-635X2000000200003

[CR20] Maiorka A, Silva AVF, Santin E, Bruno LDG, Boleli IC, Macari M (2016) Effect of broiler breeder age on intestinal mucosa development of embryos at 20 days of incubation. Braz J Poult Sci 18(2):1–4. 10.1590/1806-9061-2015-0130

[CR22] Matterson LD, Potter LM, Stutz MW, Singsen EP (1965) The metabolizable energy of feeds ingredients for chickens. Agric Exp Stn Res Rep 7:1–11. https://archive.org/details/the-metabolizable-energy-of-feed-ingredients-for-chickens

[CR24] Mesele TL (2023) Reproduction and production performance of improved chickens, production constraints, and opportunities under Ethiopian conditions. Trop Anim Health Prod 55(4):245. 10.1007/s11250-023-03524-1 37340246 10.1007/s11250-023-03653-w

[CR25] Meurer RFP, Valle FLP, Santos AS, Zanatta CP, Dahlke F, Maiorka A, Oliveira EG (2008) Interaction between breeder age and egg weight on broiler performance. Arch Vet Sci 13(3):197–203. 10.5380/avs.v13i3.11932

[CR26] Molenaar R, Stockhofe-Zurwieden N, Giersberg MF, Rodenburg TB, Kemp B, van den Brand H, de Jong IC (2023) Effects of hatching system on chick quality, welfare, and health of young breeder flock offspring. Poult Sci 102(3):102448. 10.1016/j.psj.2022.102448 36641993 10.1016/j.psj.2022.102448 PMC9846018

[CR23] Mottet A, Tempio G (2017) Global poultry production: current state and future outlook and challenges. World’s Poult Sci J 73:245–256. 10.1017/S0043933917000071

[CR27] Noy Y, Sklan D (2002) Nutrient use in chicks during the first week post-hatch. Poult Sci 81(3):391–399. 10.1093/ps/81.3.391 11902417 10.1093/ps/81.3.391

[CR28] Ramukhithi TF, Nephawe KA, Mpofu TJ, Raphulu T, Munhuweyi K, Ramukhithi FV, Mtileni B (2023) Economic sustainability and efficiency of small-scale broiler farms: A review. Sustainability 15(3):2030. 10.3390/su15032030

[CR38] R Core Team (2021) R: A language and environment for statistical computing. R Foundation for Statistical Computing, Vienna, Austria. https://www.R-project.org/

[CR29] Rostagno HS, Albino LFT, Donzele JL, Gomes PC, Oliveira RF, Lopes DC, Ferreira AS, Barreto SLT, Euclides RF (2017) Brazilian tables for poultry and swine: Feed composition and nutritional requirements, 4th edn. UFV, Viçosa, Brazil

[CR30] Rysman K, Eeckhaut V, Ducatelle R, Goossens E, Van Immerseel F (2023) Broiler performance correlates with gut morphology and intestinal inflammation under field conditions. Avian Pathol 52(4):232–241. 10.1080/03079457.2023.2215147 37132444 10.1080/03079457.2023.2201169

[CR31] Sakomura NK, Rostagno HS (2016) Research methods in monogastric nutrition, 2nd edn. FUNEP, Jaboticabal, Brazil

[CR32] Silva DJ, Queiroz AC (2002) Análise de alimentos: métodos químicos e biológicos, 3rd edn. Editora da Universidade Federal de Viçosa, Viçosa, Brazil

[CR33] Tona K, Onagbesan O, Ketelaere B, Decuypere E, Bruggeman V (2004) Effects of broiler breeder age and egg storage on egg quality, hatchability, chick quality, chick weight, and posthatch growth to forty-two days. J Appl Poult Res 13(1):10–18. 10.1093/japr/13.1.10

[CR34] Tona K, Voemesse K, N’nanlé O, Oke OE, Kouame YAE, Bilalissi A, Oso OM (2022) Chicken incubation conditions: role in embryo development, physiology, and adaptation to the posthatch environment. Front Physiol 13:895854. 10.3389/fphys.2022.895854 35677093 10.3389/fphys.2022.895854 PMC9170334

[CR35] Uni Z, Ganot S, Sklan D (1998) Posthatch development of mucosal function in the broiler small intestine. Poult Sci 77(1):75–82. 10.1093/ps/77.1.75 9469755 10.1093/ps/77.1.75

[CR36] Uni Z, Noy Y, Sklan D (1996) Developmental parameters of the small intestine in heavy and light strain chicks pre- and posthatch. Br Poult Sci 37(1):63–71. 10.1080/00071669608417837 8833528 10.1080/00071669608417837

